# Clear Cell Adenocarcinoma Arising from Endometriosis in the Groin: Wide Resection and Reconstruction with a Fascia Lata Tensor Muscle Skin Flap

**DOI:** 10.1155/2018/2139595

**Published:** 2018-04-01

**Authors:** Shozo Yoshida, Akira Onogi, Masamitsu Kuwahara, Tomoko Uchiyama, Hiroshi Kobayashi

**Affiliations:** ^1^Department of Obstetrics and Gynecology, Osaka Gyoumeikan Hospital, Osaka, Japan; ^2^Department of Plastic Surgery, Nara Medical University, Nara, Japan; ^3^Department of Pathology, Nara Medical University, Nara, Japan; ^4^Department of Obstetrics and Gynecology, Nara Medical University, Nara, Japan

## Abstract

We herein report a case of clear cell carcinoma arising from endometriosis in the groin in a 53-year-old woman. The findings of MRI and FDG/PET-CT indicated a malignant tumor, and surgical biopsy confirmed adenocarcinoma of the female genital tract. The tumor including a part of the abdominal rectus muscle and rectus sheath, subcutaneous fat, skin, and the right inguinal ligament was resected* en bloc*. The defect in the abdominal wall was reconstructed with a fascia lata tensor muscle skin flap. The tumor was composed of clear cell adenocarcinoma arising from extrapelvic endometriosis. The patient received chemotherapy with gemcitabine and carboplatin for 6 cycles and had no evidence of recurrence 7 months after the treatment. We herein described the diagnosis and surgical management of endometriosis-associated carcinoma in the groin.

## 1. Introduction

Endometriosis is a common disease in reproductive-age women, with lesions commonly being observed in the ovaries, uterine muscle layer (adenomyosis), and/or pelvic peritoneum. However, these lesions have sometimes been detected in the extrapelvic space such as the lungs, intestines, and skin [1]. The inguinal region is one of the very rare sites of extrapelvic endometriosis, with a frequency as low as 0.3–0.6% [1, 2]. Endometriosis is known to exhibit carcinogenic potential [3]. We herein present a case of a postmenopausal woman with a malignant tumor that was suspected to have arisen from endometriosis in the groin.

## 2. Case Presentation

A 53-year-old gravida 1 woman was referred to our department with a tumor in her groin. Her previous medical history consisted of endometriosis treated by surgical cautery at the age of 38 years, abdominal myomectomy for uterine leiomyoma at 39 years, and a cesarean delivery at 41 years. Although the patient entered menopause at the age of 49 years, she did not have any discomfort or pain in the inguinal region associated with menstruation before menopause. She discovered a tumor in her right groin approximately 3 years before her hospital visit. She went to a private gynecological clinic because the tumor had gradually increased in size and become painful. The gynecologist suspected a soft tissue tumor arising from muscle or adipose tissue and referred her to an orthopedist in our hospital. Surgical biopsy was performed, and the findings obtained indicated that the tumor consisted of a papillary component and was suspected of being serous adenocarcinoma based on hematoxylin and eosin (HE) staining. The tumor appeared to be adenocarcinoma, similar to metastatic ovarian cancer, and not a soft tissue tumor. The patient was referred to our department.

**Figure 1:**
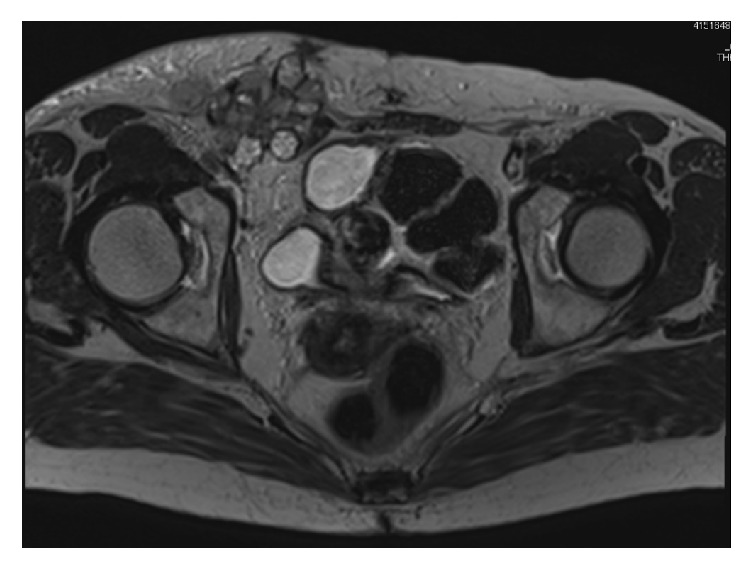
T2-weighted axial MR image, showing a multilobular mass consisting of cystic and solid components at the right inguinal site.

**Figure 2:**
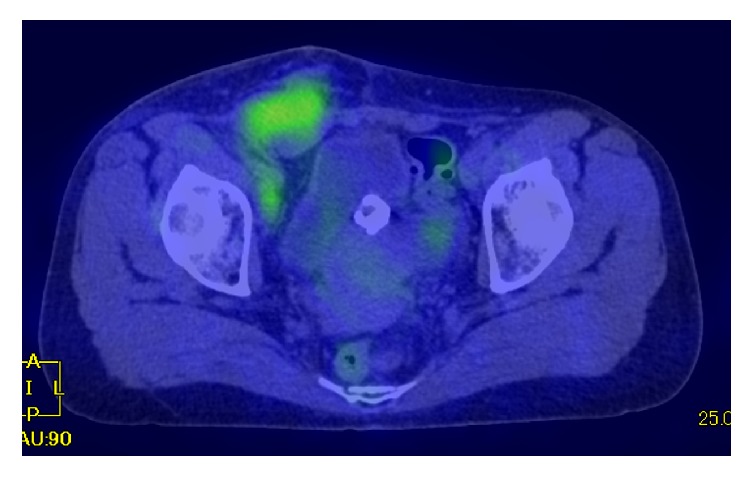
Apparent accumulation of ^18^FDG at the right groin site and right pelvic lymph nodes on FDG/PET-CT.

The tumor was palpable in the groin and had poor mobility. An ultrasound indicated that the tumor was as large as 5 cm in diameter. Magnetic resonance imaging (MRI) revealed a multilobular tumor in the right inguinal region that was 55 mm in diameter and consisted of cystic and solid components (Figure 1). ^18^F-Fluorodeoxyglucose-positron emission tomography/computed tomography (FDG-PET/CT) showed the accumulation of FDG at the tumor and right external and obturator lymph nodes, which was attributed to metastasis of the malignant tumor (Figure 2). MRI also indicated uterine leiomyoma with calcification and a right ovarian cystic tumor that appeared to be benign endometrioma because a solid part was not found in the cyst. No other lesion involving peritoneal dissemination or distant metastasis was detected. Serum CA125: 21 U/ml, CEA: 1.0 U/ml, and CA19-9: 6 U/ml were not elevated. Serum FSH: 66.2 mIU/ml and estradiol <10 pg/ml were consistent with a menopausal patient.

Since endometrioma was detected in the right ovary, the tumor was located in the groin, one of the sites of extrapelvic endometriosis, and the pathological findings of a biopsy specimen suggested serous adenocarcinoma or endometrioid adenocarcinoma, which is closely related to endometriosis, the tumor was suspected to be endometriosis-associated adenocarcinoma arising from endometriosis in the groin. Distant metastasis was not detected, and, thus, surgical resection of the tumor and lymph nodes was performed.

**Figure 3:**
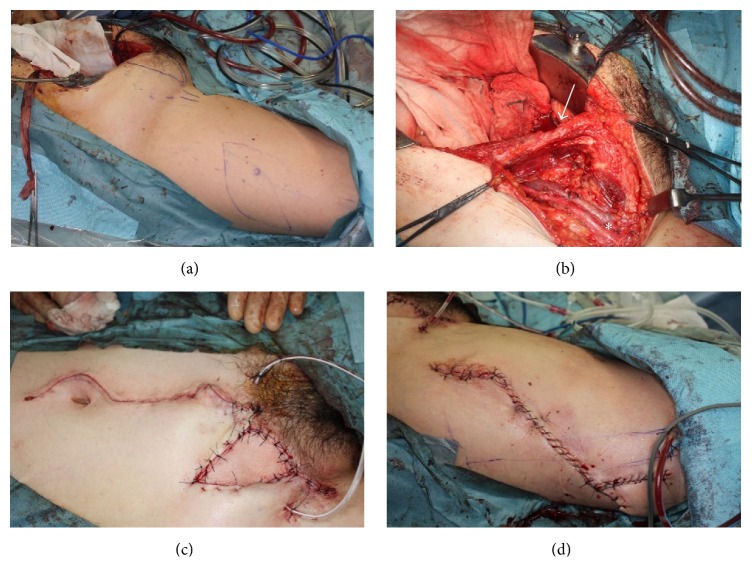
Tumor resection and reconstruction. (a) Skin markings on the right groin and femur. (b) Removal of the tumor with the abdominal wall (the arrow indicates the abdominal rectus and the asterisk the femoral vessels). (c) Reconstruction of the abdominal wall with a tensor lata muscle flap. (d) Femur site wound closure.

Abdominal hysterectomy, bilateral adnexectomy, and pelvic lymphadenectomy were initially performed, followed by a skin incision to the right lower abdomen. Skin and adipose tissue around the tumor were divided and separated as much as possible. The tumor including a part of the abdominal rectus muscle and rectus sheath, subcutaneous fat, skin, and the right inguinal ligament was resected* en bloc* with a 1.5 cm surgical excision margin. A skin incision to the right femur was performed and a skin pedicle flap was constructed. The lower abdominal wall with skin and fascia defects was reconstructed with a fascia lata tensor muscle skin flap (Figure 3).

**Figure 4:**
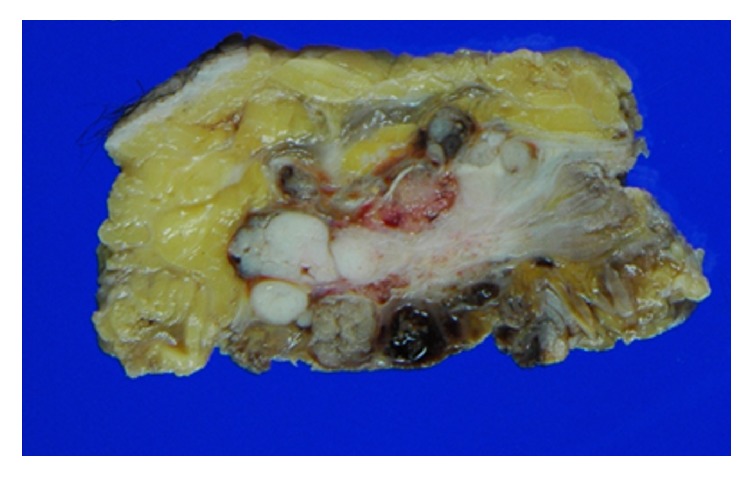
Resected tumor showing the involvement of fat tissue and the abdominal rectus.

The resected tumor was 11.5 × 9.5 × 6 cm and section surface was white. The tumor infiltrated fat tissue under the skin, inguinal ligament, and abdominal rectus (Figure 4).

Macroscopic findings showed that the tumor mainly grew in the cystic component and partially expanded outside of the cyst. Erosion, bleeding, and the accumulation of hemosiderin were observed in the cyst wall, while a small spindle cell-like endometrial stroma was detected below the cyst wall. Immunohistostaining revealed that estrogen receptors (ER) were positive in the epithelium and stroma of the cyst wall, while CD10 was positive in the stroma, suggesting that the cyst was an endometriotic cyst.

**Figure 5:**
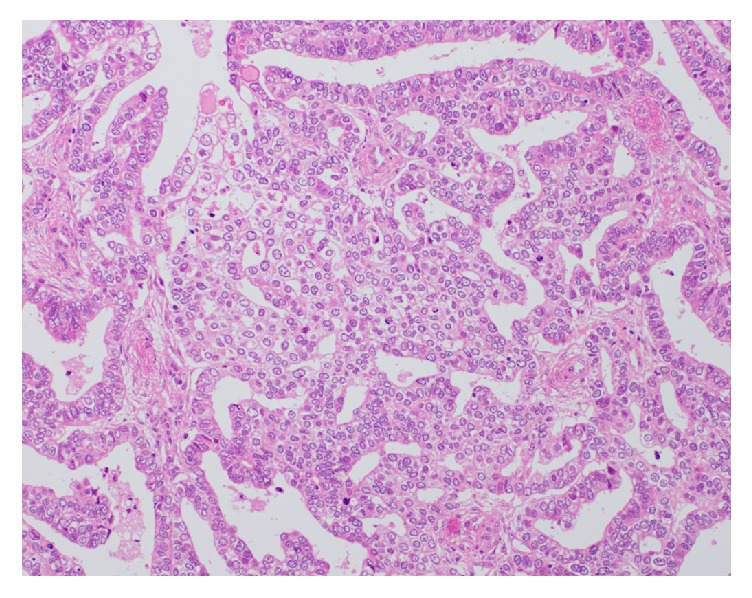
Microscopic findings of an excised specimen of the tumor. A clear cytoplasm and round-shaped dense nuclei with severe atypia, suggesting cell adenocarcinoma (hematoxylin and eosin stain X200).

The tumor showed papillary, tubular, and cribriform growth in the fibrovascular stroma of the cyst. Tumor cells had a clear and eosinophilic cytoplasm with a round-shaped nucleus with nuclear atypia. Immunohistostaining showed that hepatocyte nuclear factor-1beta (HNF-1*β*) was positive, p53 was negative, ER was negative, and progesterone receptor (PgR) was focally positive, indicating that most of the tumor presented the features of clear cell adenocarcinoma (Figure 5).

The tumor also invaded outside the cyst, in which an alveolar tumor with high-grade nuclear atypia grew in vesicular and sheet forms. Immunohistostaining of the tumor at this site showed that HNF-1*β* was negative, p53 was positive, ER was partially positive, and PgR was negative, indicating poorly differentiated adenocarcinoma. The tumor also involved 8 out of the 61 resected lymph nodes. Although endometriosis was detected in the right ovary and broad ligament of the uterus, no other malignant lesions were found in the abdominal cavity. Furthermore, the cytology of ascites was negative.

Based on the pathological findings of (1) benign endometriotic cysts in the cystic lesion, (2) endometrial stroma around the cystic lesion, (3) transition from benign to malignant features in the epithelium of the cyst wall, and (4) no other primary lesion, the tumor was consistent with clear cell adenocarcinoma arising from extrapelvic endometriosis

After the surgical wound had healed, we recommended chemotherapy with carboplatin and paclitaxel, which are commonly used in the treatment of ovarian cancer. The patient agreed with our proposal, but developed severe hypersensitivity to paclitaxel; therefore, gemcitabine and carboplatin were alternatively prescribed for 6 cycles. Although the patient developed right leg edema, she remained healthy and had no evidence of recurrence 20 months after the treatment.

## 3. Discussion

Sampson first described inguinal endometriosis in 1925 [4], and many reports have been since published on this condition [5]. Inguinal endometriosis is considered to account for 0.3–0.6% of all cases of endometriosis [1, 2, 5], with lesions commonly occurring in the extraperitoneal part of the round ligament. Although the right inguinal region is a major site of inguinal endometriosis, as in the present case, the reason for this currently remains unknown [2]. In most cases, patients develop symptoms such as painful induration in the groin region related to menstruation, which may go unnoticed for small lesions. Our patient was postmenopausal and did not have any pain or discomfort in this groin region before menopause.

As Sampson also reported for carcinoma arising from endometrioma of the ovaries in 1925 [3], endometrial tissue exhibits carcinogenic potential, with the incidence of carcinogenesis being estimated as less than 1.0%. [6]. A large number of reports have described carcinomas associated with extrapelvic endometriosis, such as the lungs, intestines, and diaphragm [7–10]. To date, there have only been 7 cases (6 reports) of carcinoma arising from inguinal endometriosis [11–15], and, thus, our case appears to be very rare. The most frequent symptom was the palpation of a tumor in the groin.

MRI and FDG/PET-CT were applied to diagnose the tumor in the present case. Diffusion-weighted images and contrast-enhanced images by MRI as well as the accumulation of FDG in the tumor on FDG-PET-CT were useful for identifying the malignant tumor. These imaging examinations and pathological findings contributed to the tumor being diagnosed as a malignant tumor arising from extrapelvic endometriosis before radical surgery.

Since endometrial tissue was clearly observed near the carcinoma, the tumor was diagnosed as endometriosis-associated adenocarcinoma. Some types of pathologies have been reported for endometriosis-associated carcinoma including clear cell, endometrioid, and serous adenocarcinomas [16]. The most common histological type of endometriosis-associated carcinoma in the groin is clear cell adenocarcinoma [15].

A treatment has not yet been established for carcinoma arising from extrapelvic endometriosis. We previously discussed therapeutic strategies at a conference. Since the tumor developed in the groin and involved the pelvic lymph nodes with no evidence of para-aortic nodule metastasis using imaging modalities, we performed abdominal hysterectomy, bilateral adnexectomy, pelvic lymphadenectomy, tumor resection in the groin, and reconstruction of the abdominal wall instead of typical staging surgery for ovarian cancer. We also performed these procedures in cooperation with a plastic surgeon. In order to remove the tumor with an adequate margin, the tumor was radically resected with a part of the abdominal rectus muscle and rectus sheath, fat layer under the skin, skin, and the right inguinal ligament. A skin flap was used with a penetrator from the lateral femoral circumflex artery from the tensor fascia latae muscle and fixed to the rectus abdominis muscle and abdominal external oblique muscle through a subcutaneous tunnel in order to reconstruct the abdominal wall. This technique is sometimes employed to reconstruct large abdominal defects [17]. To the best of our knowledge, this is the first case in which endometriosis-associated carcinoma in the groin was resected and reconstructed using this method. The clinical course after surgery was good, indicating that this method is applicable to similar cases.

It currently remains unclear whether adjuvant chemotherapy is suitable for extrapelvic endometriosis-associated carcinoma. After the treatment of epithelial ovarian cancer, adjuvant chemotherapy was initiated because of pelvic lymph node metastasis. Paclitaxel and carboplatin were prescribed, but the patient developed serious hypersensitivity to paclitaxel. Thus, gemcitabine and carboplatin were administered for 6 cycles. Although slight right leg edema was observed, the patient remained healthy with no evidence of recurrence 9 months after the completion of therapy.

Although difficulties were associated with diagnosing and selecting a therapeutic strategy for this patient because of the rarity of the tumor, we successfully treated her by employing precise imaging examinations and cooperating with a plastic surgeon. Extrapelvic endometriosis-associated carcinoma needs to be considered in postmenopausal women with tumors in the groin region.
